# Differences in enhancer activity in mouse and zebrafish reporter assays are often associated with changes in gene expression

**DOI:** 10.1186/1471-2164-13-713

**Published:** 2012-12-19

**Authors:** Ana Ariza-Cosano, Axel Visel, Len A Pennacchio, Hunter B Fraser, José Luis Gómez-Skarmeta, Manuel Irimia, José Bessa

**Affiliations:** 1 Centro Andaluz de Biología del Desarrollo (CABD), CSIC-Universidad Pablo de Olavide-Junta de Andalucía, Ctra. Utrera Km 1, Seville, 41013, Spain; 2 Genomics Division, Lawrence Berkeley National Laboratory, Berkeley, CA, 94720, USA; 3 U.S. Department of Energy Joint Genome Institute, Walnut Creek, CA, 94598, USA; 4 Department of Biology, Stanford University, Stanford, CA, 94305, USA; 5 The Donnelly Centre, University of Toronto, 160 College Street, Toronto, Ontario, M5S 3E1, Canada

**Keywords:** Evolution, Vertebrates, Trans-changes, Trans-evolution, Enhancers

## Abstract

**Background:**

Phenotypic evolution in animals is thought to be driven in large part by differences in gene expression patterns, which can result from sequence changes in *cis-*regulatory elements (*cis-*changes) or from changes in the expression pattern or function of transcription factors (*trans-*changes). While isolated examples of *trans-*changes have been identified, the scale of their overall contribution to regulatory and phenotypic evolution remains unclear.

**Results:**

Here, we attempt to examine the prevalence of *trans-*effects and their potential impact on gene expression patterns in vertebrate evolution by comparing the function of identical human tissue-specific enhancer sequences in two highly divergent vertebrate model systems, mouse and zebrafish. Among 47 human conserved non-coding elements (CNEs) tested in transgenic mouse embryos and in stable zebrafish lines, at least one species-specific expression domain was observed in the majority (83%) of cases, and 36% presented dramatically different expression patterns between the two species. Although some of these discrepancies may be due to the use of different transgenesis systems in mouse and zebrafish, in some instances we found an association between differences in enhancer activity and changes in the endogenous gene expression patterns between mouse and zebrafish, suggesting a potential role for *trans-*changes in the evolution of gene expression.

**Conclusions:**

In total, our results: (i) serve as a cautionary tale for studies investigating the role of human enhancers in different model organisms, and (ii) suggest that changes in the *trans* environment may play a significant role in the evolution of gene expression in vertebrates.

## Background

The idea that phenotypic evolution occurs mostly by changes in gene expression rather than in protein coding sequences has been gaining increasing acceptance [1-3]. However, how the expression of a particular gene evolves between lineages is less clear. Gene expression is controlled by sets of regulatory sequences (*cis-*regulatory elements) that are recognized by protein transcription factors (*trans-*factors) differentially expressed among cell types and developmental stages. The interplay between *cis-*elements and expressed *trans-*factors determines gene expression; thus, the evolution of gene expression can occur due to changes in either the *cis-*regulatory elements (hereafter referred to as “*cis-*changes”) or in the associated *trans* environment (“*trans-*changes”, which can be due to changes in the expression of the *trans-*factors themselves, or in their activity, specificity, etc.).

The relative contribution of *cis* and *trans-*changes to the evolution of transcriptional regulation is not well understood, in particular in the vertebrate lineage. Several studies have addressed this question comparing closely related lineages in other systems (e.g. yeasts [4-9] or Drosophilas [10,11]). In general, these studies show that, at short evolutionary distances, *cis*-changes are the dominant force shaping gene transcriptional regulation. Although less information is available on vertebrate models, a similar conclusion was reached by Wilson and co-workers [12], demonstrating that for genes of the human chromosome 21, most of expression divergence between mice and human was associated to *cis*-changes. Nevertheless, the difficulty of experimental study in vertebrate models and the presence of much more complex *cis*-regulatory landscapes in these organisms make it currently impossible to confidently identify and compare the full *cis*-regulatory complement of most vertebrate genes in different species. A way to circumvent this problem has been to focus on the evolution of highly conserved non-coding elements (CNEs, [13-16]) associated to certain genes, for which orthology relationships between species can be confidently established. Functional assays of these CNEs showed that they often act as transcriptional enhancers, harboring a substantial part of the information on when, where and how much a gene must be transcriptionally active [14,15,17-19]. Thus, CNE comparisons allow a partial evaluation of the contribution of *cis* vs. *trans* changes between species. Along these lines, different groups have compared the behavior of a handful of orthologous CNEs near key developmental genes in their respective native hosts, often mouse and zebrafish, finding that only around a third of the orthologous enhancers may drive different developmental expression [20-24]. In these studies, however, it is not possible to tease apart the contribution of *cis* vs *trans* changes in these differences. In a recent study, Ritter and co-workers compared the expression driven by 13 human CNEs in mouse and zebrafish transgenic embryos, finding that 5/13 (39%) CNEs showed different expression in the two hosts [25], suggesting differences in the *trans* environment, consistent with previous results [19]. However, the low number of reported cases makes it difficult to infer the real extent of *trans* effects in the evolution of gene expression.

Here, we have aimed to directly test the potential impact of *trans-*changes in the evolution of enhancer activity and of endogenous gene expression. As a proxy for potential differences in *trans* environments, we have compared the enhancer activity of 47 human enhancers in mouse and stable zebrafish transgenic embryos. We have found that the majority of the sequences tested (83%) show differences in reporter expression. Despite possible confounding experimental factors, many of these differences are likely to be caused by changes in the *trans* environment between the two distantly related vertebrate species. The frequency of such differences was high for both ancestral CNEs that are conserved in zebrafish and those that diverged beyond recognition in the teleost, representing CNEs with mild and severe *cis* changes, respectively. In addition, four-way comparisons of CNE-driven and associated endogenous gene expressions in zebrafish and mouse showed that the majority of observed changes in gene expression (8 out of 11 cases) are in agreement with a *trans* change, suggesting that *trans*-changes may play a role in driving these differences.

## Results

### Widespread differences in enhancer activity between mouse and zebrafish

**Figure 1:**
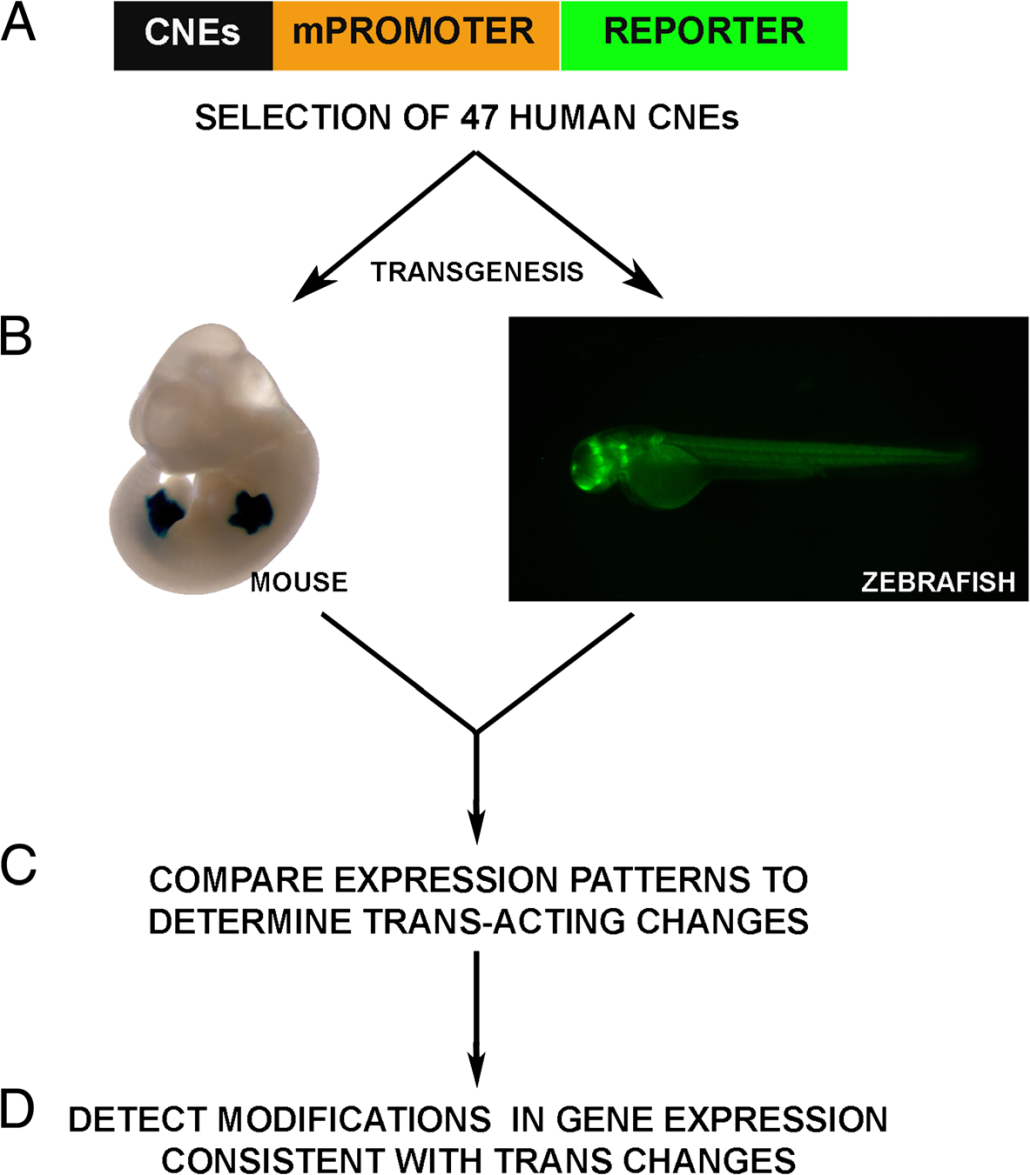
**Experimental workflow.** A subset of 47 conserved non-coding elements (CNEs, [26]) were randomly selected (**A**), and tested for enhancer activity using transgenesis in zebrafish and mice (**B**). Transgenic expression was decomposed into major homologous anatomical terms, and systematically compared between mouse and zebrafish embryos to identify cases of differences in *trans* environments (**C**). Finally, 26 of these CNEs could be associated to putative target genes, for which endogenous gene expression data were gathered to detect changes in gene expression between zebrafish and mouse that were consistent with *trans-*changes between the two species (**D**).

**Figure 2:**
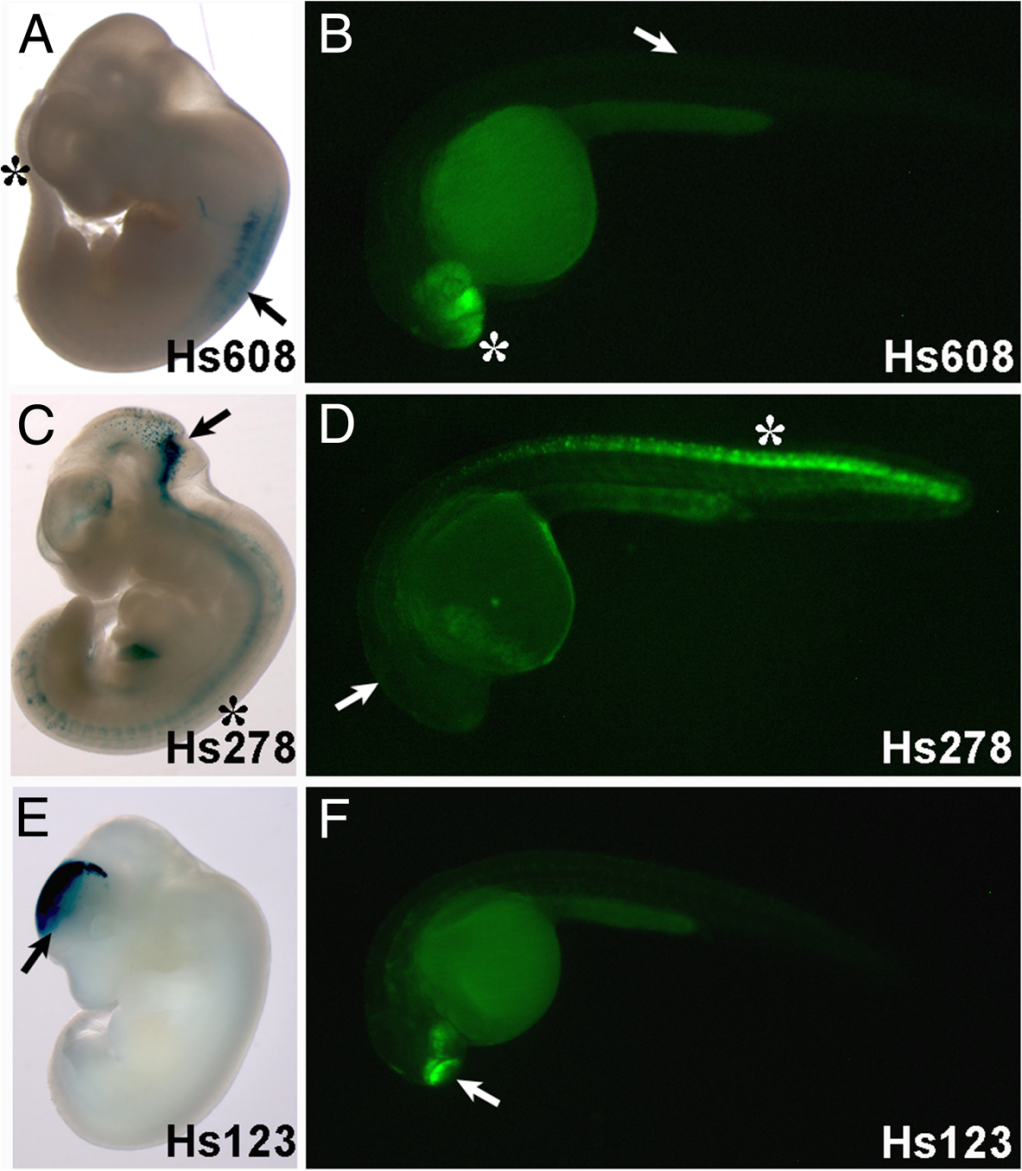
**Comparison of enhancer activity of different CNEs in mice and zebrafish.** A, B) Expression driven by the Hs608 enhancer shows mouse-specific expression (**A**) in the dorsal root ganglia and spinal cord (arrow), and zebrafish-specific expression (**B**) in the forebrain (asterisk). C, D) The Hs278 enhancer drives expression in hindbrain (arrow) and spinal cord (asterisk) in mouse (**C**) but only in spinal cord in zebrafish embryos (**D**). E, F) The Hs123 enhancer drives similar expression in the forebrain of mouse (**E**) and zebrafish (F, arrow).

In order to assess the potential extent of evolutionary *trans-*changes between teleosts and mammals we compared the enhancer activity of 47 sequences in mouse and zebrafish. We used a subset of randomly selected human CNEs that were previously shown to have enhancer activity in mouse 11.5dpc (days post coitum) embryos [26]. These exact same sequences were used to generate stable zebrafish transgenic lines driving GFP reporter expression (Figure 1), which produce much more robust results and avoid phenotypic variation typically associated with transient transgenic assays in zebrafish [27]. GFP expression was monitored in F1 transgenic embryos, from 24 to 48 hours post-fertilization (hpf), a developmental range that includes the stage homologous to the mouse 11.5dpc stage (the phylotypic stage [28]). Consistent GFP expression from multiple founders for each CNE was then decomposed and annotated into major anatomical domains homologous to those used for mouse embryos. Although many of the CNEs tested have shown consistent expression between mouse and zebrafish for at least one anatomical domain (39 out of 47), many discrepancies of expression were also detected, resulting in a surprisingly high number of sequences that presented at least one major species-specific anatomical domain (39 out of 47; 83%, containing on average 2.4 expression domains in zebrafish and 2.3 in mouse per CNE; Additional file 1). Since the sequences probed were identical in both species, these divergences could result from the specific settings used for the mouse and zebrafish reporter assays (see discussion) and/or to differences between the transcription factor environments of developing mouse and zebrafish embryos, which may be interpreting the same *cis-*regulatory information differently. In the most extreme cases, 17/47 (36%, containing on average 2.2 expression domains in zebrafish and 1.9 in mouse per CNE) of these sequences showed dramatically different patterns (divergence of expression for 75% or more anatomical domains). One example of such global differences is the enhancer Hs608, which shows activity in dorsal root ganglia and spinal cord in mouse, but only forebrain expression in zebrafish (Figure 2A and B). In other cases the divergence was less extensive, often with few extra expression domains in mouse and/or zebrafish. For example the Hs278 enhancer drives expression to the hindbrain and spinal cord in transgenic mice (Figure 2C), whereas transgenic zebrafish have only spinal cord expression (Figure 2D). It should be noted, however, that some of these differences may also be due to the use of a different transgenesis system for each species (see Discussion). Finally, only 8/47 (17%, containing on average 2.3 expression domains in zebrafish and 2.3 in mouse) sequences showed fully consistent reporter expression patterns (Additional file 1). This is the case of the Hs123 enhancer, which shows a shared expression in mice and zebrafish in the forebrain (Figure 2E and F).

### Differences in enhancer activity are frequent in ancestral CNEs with either severe or mild *cis-*changes

One interesting hypothesis is whether *trans-*changes may correlate with changes in the sequences to which they bind; in other words, they may correlate with *cis-*changes. This may happen, for instance, if an enhancer loses the binding site for a specific *trans* factor, relieving the selective pressure to keep this *trans* factor expressed in the corresponding tissue. The opposite may also be true: loss of a *trans* factor’s expression in a domain may relieve the selective constraint to preserve the corresponding binding sites in the enhancer sequence. To assess this possibility, we selected a group of phylogenetically comparable CNEs present in the gnathostome ancestor (i.e. conserved to the shark *Callorhinchus milli*, 37 aCNEs, see Methods) and divided them into two major groups, whether they have been conserved (20 CNEs) or diverged beyond recognition/lost (17 CNEs) in the lineage of zebrafish. Ancestral sequences that cannot be detected in zebrafish are expected to have evolved at a much faster rate or been fully deleted (severe *cis-*changes); in contrast, those CNEs that can be detected in fish are more likely to have experienced less evolutionary sequence change (mild *cis-*changes). Consistent with this idea, phyloP scores show a nearly 50% higher average rate of evolution within placental mammals for those aCNEs not detectable in zebrafish (0.159 vs. 0.107, p = 0.042, ANOVA test). Similarly, the ratio between positions with low phyloP score (score < −1, accelerated evolution) and high score (score > +1, positions under strong purifying selection) is 2.5 times higher for the CNEs absent in zebrafish (p < 0.0001, Chi-square test). Conversely, the average sequence conservation among mammals, as measured by PhastCons scores, is higher for those CNEs present in the zebrafish genome (0.699 vs. 0.794, p = 0.047). Finally, the alignment of the orthologous CNEs from human, mouse, chicken and Xenopus show higher average conservation for CNEs conserved in zebrafish (63.9% vs. 70.0%, p = 0.043).

However, despite their different evolutionary sequence history, analyses of each group separately showed that species-specific domains of enhancer activity are very frequent in both types of CNEs, with either severe or mild changes (82% and 90% respectively, p = 0.863; Additional file 2). Similarly, the fraction of CNEs with global reporter expression changes is similar (8/17 vs. 5/20), and 3/5 of the CNEs with identical expression patterns correspond to aCNEs with severe *cis-*changes. Since each CNE may drive reporter expression to one or more anatomical annotations, we have also quantified the percentage of the affected expression domains per CNE. Here, we also found a high proportion of affected domains in both groups, with a slightly higher (though not significantly) representation of species-specific domains in the non-conserved/lost group (60% vs 47%, p = 0.09, Fisher 1-tail test; Additional file 2). These results, although not providing evidences against a major role of *cis*-changes in the control of gene expression in evolution, suggest that the effect of the *trans* environment (and/or transgenesis system employed) may be important for CNE function, regardless of the extent of their *cis*-regulatory sequence divergence.

### Differences in enhancer activity are associated with changes in gene expression

**Figure 3:**
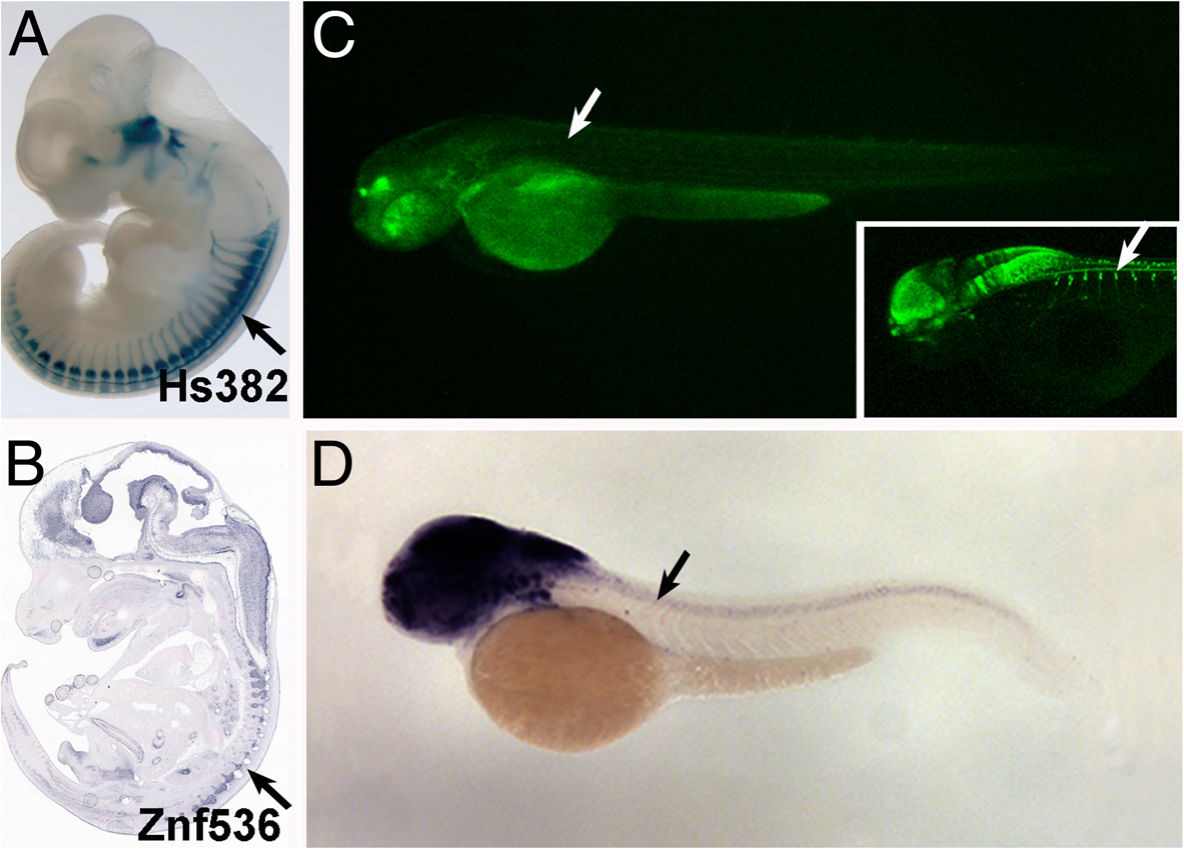
**Examples of a change in endogenous gene expression associated to different enhancer activities in mouse and zebrafish.A**) Expression driven by the Hs382 enhancer is detected in DRG (arrow) in mouse. **B**) This expression is coincident with the Hs382 target gene, *Znf536* (arrow). **C**) In zebrafish, the Hs382 enhancer does not drive expression in DRGs (arrow). This contrasts with a positive control for DRG expression (inset, arrow; Tg(−3.1neurog1:GFP)sb2). **D**) This absence coincides with lack of expression of *Znf536* in DRG (arrow).

We have shown that many CNEs have divergent behaviors in mouse and zebrafish embryos, which could be due to possible differences in the associated *trans* environments in the two species in at least some instances. In this case, however, this would not imply that these putative *trans-*changes have played a role in the evolution of the associated endogenous gene expression patterns in the two species. To assess the potential evolutionary importance of the observed differences in enhancer activity, we next compared the expression patterns of the genes associated with the studied CNEs. We combined synteny and comparisons of expression patterns between gene and enhancer to confidently associate genes to 26 CNEs (see Methods). For these genes, expression data were generated or surveyed in available databases for both zebrafish and mouse at comparable time points. Then, we annotated the same anatomical terms as per the enhancer assays, which allowed 4-way comparisons: zebrafish endogenous gene expression (Zg), mouse endogenous gene expression (Mg), zebrafish transgenic reporter expression (Zt) and mouse transgenic reporter expression (Mt) (Additional file 3). Despite the large fraction of differences in enhancer activity, only 11/26 genes showed changes in major expression domains between the two species at the studied developmental stages (Table 1). Intriguingly, however, the CNEs associated to these 11 genes show a higher fraction of divergent expression domains in the reporter assays (an average of 67% of expression domains diverge between mouse and zebrafish) than the remaining 15 genes (average of 38%; p = 0.012, T-test; Additional file 3). Moreover, the majority (8/11, 73%) of the CNEs associated with the divergent genes show consistent changes in enhancer activity for at least one of the divergent anatomical terms (Table 1). One example of this is the enhancer Hs382, which drive reporter expression in dorsal root ganglia in mouse consistent with the associated gene, *znf536* (DRG; Figure 3A and B), whereas both the reporter and the target gene are absent from this structure in zebrafish (Figure 3C and D). Therefore, these results suggest that some differences in enhancer activity between mouse and zebrafish are indeed mirroring changes in the endogenous gene expression patterns.

**Table 1:** CNEs associated to target genes that show changes in expression between zebrafish and mice

**CNE**		**Mt**	**Zt**	**Mg**	**Zg**	**Trans&Gene**	**NoTrans&Gene**
**Hs259**	Limbs	+	-	+	-	Yes	
	Spinal cord	-	+	+	+		
**Hs327**	Hindbrain	+	+	+	+		
	Forebrain	-	+	+	+		
	Spinal cord	+	+	+	-		Yes
	Midbrain	-	+	+	+		
**Hs382**	Forebrain	+	+	+	+		
	Somites	+	+	+	+		
	Spinal cord	+	-	+	+		
	DRG	+	-	+	-	Yes	
	Eye	-	+	+	+		
	Notochord	-	+	+	+		
	Hindbrain	+	-	+	+		
	Midbrain	+	-	+	+		
**Hs422**	Forebrain	+	+	+	+		
	Spinal cord	-	+	-	-		
	Nose	+	-	+	-	Yes	
	Hindbrain	-	+	-	-		
**Hs595**	Forebrain	+	+	+	+		
	Midbrain	+	-	+	+		
	Nose	+	-	+	-	Yes	
**Hs609**	Forebrain	+	-	+	+		
	Limbs	+	-	+	-	Yes	
**Hs671**	Forebrain	+	+	+	+		
	Eye	-	+	+	+		
	Hindbrain	-	+	+	+		
	DRG	-	+	+	-		
**Hs672**	Forebrain	+	-	+	+		
	Midbrain	+	+	+	+		
	Spinal cord	+	-	+	-	Yes	
**Hs687**	Forebrain	+	+	+	+		
	Spinal cord	+	-	+	-	Yes	
**Hs774**	Hindbrain	+	+	+	+		
	Limbs	+	+	+	-		Yes
	Forebrain	-	+	+	+		
	Eye	-	+	-	+	Yes	
**Hs1114**	Midbrain	+	+	+	+		
	Hindbrain	+	+	-	+		
	Spinal cord	+	+	+	+		

Presence (+) or absence (−) of expression for the transgene in mice (Mt), transgene in zebrafish (Zt), mice target gene (Mg), and zebrafish target gene (Zg). “Trans & Gene” refers to changes in the expression of target genes consistent with *trans-*changes and “No Trans & Gene” indicates changes in the expression of target genes coincident with no *trans* events. DRG - Dorsal root ganglia.

## Discussion

We have compared enhancer activity of 47 human conserved non-coding sequences between mouse and zebrafish. Surprisingly, we found that the vast majority of these sequences (39/47, 83%) show discrepancies in at least one expression domain between mouse and zebrafish, indicating a remarkably different behaviour of the same enhancer sequences when tested in these two distantly related vertebrate lineages. Since the sequences tested are the same in the two transgenesis systems, it is possible that differences in reporter expression patterns may correspond to differences in the cellular TF environment of each species. A fraction of these differences may also result from the use of different transgenesis systems in each species; however, as discussed below, these are not likely to account for the majority of cases (see below).

### Differences in enhancer activity: trans-changes or experimental differences between transgenesis systems?

A major question raised by our results is whether the vast differences are mostly due to differences in *trans* environments between both species or to experimental differences between the two transgenesis systems, or a similar combination of both. The experimental differences between mouse and zebrafish include the use of different minimal promoters (*hsp68* vs *gata2a*), reporter genes (LacZ vs GFP), transgenesis techniques (pronuclear injection vs Tol2 transposon) and endogenous characteristics associated to each animal model (opaque vs transparent embryos). To ensure the reproducibility of results we have performed transgenesis in zebrafish using the ZED vector [29] and analysed reporter expression in stable transgenic embryos (F1 generation). This vector is able to minimize the position effect usually associated to transposon mediated zebrafish transgenesis. In addition, this vector contains the *gata2a* minimal promoter, which has been has been shown to be able to read a wide range of enhancers in enhancer assays [19,27,30-40]. A similar performance for this minimal promoter was observed in a large-scale enhancer trap screen, being able to recapitulate the expression pattern of genes nearby the enhancer trap insertions with minimal noise [41]. Similarly, the mouse *hsp68* minimal promoter has been extensively used by many different research groups, for a broad range of tissue-specific enhancers [26,42-50]. Tol2 transposon mediated transgenesis [51] is broadly used in zebrafish because it is very efficient, usually resulting in the integration of single to few copies of the reporter construct. This contrasts with the integration of multiple concatenated copies generated by the pronuclear injection of linearised DNA, widely applied in mouse transgenesis. Multiple copies of the reporter construct might reduce the position effect, but the consequent over-sensibility of the system might be a concern. The ability to control the enzymatic dependent revelation of the reporter gene (LacZ) in mouse reporter assays, together with the endogenous opacity of mice embryos might compensate this over-sensibility, making it a system possible to compare with the zebrafish reporter assay. Therefore, although, we cannot rule out that a fraction of the differences observed in this analysis are due to the individual features of the vectors, these are unlikely to account for the majority of the large number of differences in enhancer activity observed in our study (this would imply that most published data for enhancer activity in mouse and zebrafish is also not reliable). Furthermore, several observations from our results suggest that experimental considerations may indeed be behind only in a minority of cases: (i) A similar number of anatomical domains are detected in both enhancer activity assays (106 in mouse and 113 in zebrafish), thus “sensibility” of both assays is similar. (ii) Differences in enhancer activity due to ectopic expression in one of the species represent a comparable percentage of the total and are similarly distributed across anatomical domains (45 (54%) versus 38 (46%) cases in zebrafish and mouse, respectively; domain distribution in Additional file 4). (iii) Several differences in enhancer activity are coincident with changes in putative target genes.

Finally, several considerations were taken in order to minimize differences in enhancer activity that could be due to differences in the development and/or body plan of the two species: (i) Only wide expression in major conserved anatomical terms was considered (e.g. “hindbrain”, “spinal cord”, etc.; see Methods) to avoid cell type- and subdomain-specific differences. (ii) The comparisons were performed in homologous developmental stages corresponding to the vertebrate phylotypic stage, which shows the highest similarity between species during embryonic development, both morphologically and transcriptomically [28]. (iii) Taking advantage of the *in vivo* nature of the zebrafish transgenic assays, we monitored GFP expression between 24 and 48hpf – which encompasses the most likely equivalent of the mouse 11.5dpc embryo – to minimize differences due to highly dynamic expression patterns. (It should be noted, however, that differences in the time of onset of expression of a particular TF in a given developmental domain is in itself a true *trans* change, with potentially important implications for the expression of the endogenous gene).

In summary, these considerations indicate that at least a significant fraction of the observed differences in reporter expression between mouse and zebrafish may correspond to changes in the *trans* environments (in particular those cases associated to changes in the endogenous gene expression), either by differences in the location and/or time of expression of *trans* factors in both species.

### Discrepancies with previous studies in vertebrate lineages

Ritter and co workers estimated that ~39% of enhancers (5/13) may show differences in expression between mouse and zebrafish [25], about half of the frequency we report here. However, the main focus of Ritter and colleges’ work was the study of *cis* evolution, and therefore they investigated only a limited number of cases of *trans* evolution; indeed, Ritter et al’s and our results are not statistically different (p = 0.247, Chi-squared test). In addition, to avoid the high degree of mosaicism and variability associated with unstable transgenesis in zebrafish [27], we have performed the present study generating several stable transgenic lines for each of the studied CNEs. This approach allows more confident identification of expression domains, in particular in the case of very restricted domains or if the enhancer activity is mild or not very robust in that particular region. A clear example of such effect is observed in the Hs200 enhancer. Transient transgenic embryos always present a consistent strong expression in the forebrain (Additional file 5, and [29]), also observed in our four independent stable transgenic lines for this CNE. However, the stable lines showed additional – weaker, yet consistent – expression in the midbrain, hindbrain and spinal cord (Additional file 5). Therefore, our results, obtained using stable transgenic lines, are likely to be more sensitive than previous studies using transient transgenesis. In Additional file 6, we summarize other previous studies comparing enhancer activity in mouse and zebrafish. From these, 13/20 (65%) compared cases show divergent expression patterns between mouse and zebrafish, further supporting the existence of a large number of trans changes between both species [19,21,26,27,31,43,52-55].

Finally, it should be noted that ours and other studies in vertebrates systems differ in a substantial point with those performed in other model organisms such as yeast and Drosophila [4,6-11]. In the latter, differences in gene expression were usually estimated as variation in relative quantitative expression, whereas in vertebrate systems only differences in spatiotemporal expression (i.e. anatomic domains at certain developmental stages) are assessed. For this reason, these studies complement each other investigating the outcome of different gene expression traits during evolution.

### Changes in *trans* environments and the evolution of gene expression

In addition to a survey for differences in enhancer activity between zebrafish and mouse, we have observed that the genes that present changes in expression patterns are associated to enhancers that drive more divergent reporter expression between zebrafish and mouse. Indeed, eight of such cases of differences in enhancer activity readily correspond with the changes in the expression of the endogenous genes. It is therefore tempting to speculate that these differences in enhancer activity (and thus potentially *trans-*changes) are, at least in part, responsible for the evolutionary differences in the expression of these endogenous genes between the two studied vertebrate lineages, with potential biological consequences. Remarkably, despite the low number of cases, we find that most genes (73%) that show differences in expression patterns between fish and mammals have an associated difference in enhancer activity, suggesting that this may be a more widespread phenomenon than commonly assumed, at least for CNE-associated genes.

Another intriguing finding was the large proportion of genes that did not show differences in expression patterns in zebrafish and mouse despite their putative enhancers behave differently. The simplest explanation for this observation may be that these discrepancies in enhancer activity are due to the experimental differences discussed above. However, other biological explanations are also plausible. For instance, it is conceivable that there is a different need in the two species for other partially redundant or additive regulatory elements, not present in the tested CNE, to fully respond to the specific *trans* environment. In support of this idea, most *trans-*changes that were not associated with changes in gene expression were due to the lack of expression domains in the transgenic embryos for any of the species (14/15 in mouse and 13/16 in zebrafish, as determined by the 4-way comparisons, Additional file 3). Another, non-mutually exclusive, explanation may be the co-evolution between the enhancers and the *trans* environments in the different lineages, or *cis-trans* compensation. In line with this hypothesis, recent reports in *Drosophila* and mouse provide evidence that this compensation may be larger than generally considered [56,57]. Consistently, Ritter and co workers found a better correlation for enhancer activity when testing zebrafish and human ortholog sequences in zebrafish and mice, respectively, than both orthologs in zebrafish [25].

## Concluding

Given the large number of differences in enhancer activity detected in this study, our results suggest that changes in *trans* environments may be more common in vertebrate evolution than previously anticipated, and that some of these changes may be associated with evolution of endogenous gene expression. Future research should focus on identifying the TFs responsible for *trans-*changes, and which evolutionary differences exist in those TFs (changes in gene expression caused by *cis* and/or *trans* mutations, variations in activity, specificity, etc) that are ultimately responsible for the evolution of gene expression. Finally, our results serve as a strong cautionary tale for studies investigating the regulatory function of human sequences in classical vertebrate model organisms, whether the observed variation in enhancer activity is due to specifics of each transgenesis technique, or to true biological differences in *trans* environments across large evolutionary time-scales.

## Methods

### Functional reporter analyses in zebrafish and comparison with mice reporters

Human CNEs, previously shown to have enhancer activity in mouse [26], were randomly selected, isolated from human genomic DNA and subcloned in a standard Gateway entry vector (pENTR/D-TOPO vector, Invitrogen). Sequences were transferred by recombination to a Gateway compatible reporter vector (the ZED vector) and zebrafish transgenesis was performed as described [29]. Stable transgenic lines were isolated and GFP expression was assessed at 24 and 48 hours post fertilization (hpf) from embryos reared at 28°C and staged according to standard protocols [58]. GFP was observed and documented using a stereomicroscope fitted for epifluorescence (SMZ 1500, Nikon) with a digital camera attached (F View II, Olympus). At least 3 independent stable transgenic lines were analyzed per CNE (with the exception of Hs799 for which only 2 lines were analyzed), and expression patterns were defined by presence of the reporter gene (GFP) in at least half of these lines (Additional files 7 and 8). Mice reporter data was extracted from the “VISTA enhancer browser” database ([26]http://enhancer.lbl.gov/). Extra annotations were added when a tissue was considered positive in zebrafish and expression was observed in mice in half or more cases of the available data (Additional file 7). Comparison was only performed for anatomical regions present in both species. Five enhancers previously tested in zebrafish by Lee and coworkers ([59]; Hs702, Hs901, Hs1043, Hs1114 and Hs1358) were included in the data set for mice and zebrafish comparison. Expression domains were compared by analyzing mouse expression at a single time point (11.5 days) and two corresponding developmental time points (24 and 48hpf) in zebrafish. This comparison is summarized in Additional files 1 and 2. The transgenic line Tg(−3.1neurog1:GFP)sb2, that shows expression in the dorsal root ganglia, has been previously reported [60,61].

### Sequence conservation analyses

Conservation of CNEs in the elephant shark *C. milli* was obtained from Lee et al. [2011]. To complement and update this analysis, all studied CNEs were blasted against the most up-to-date *C. milli* trace databases (both Sanger and 454 reads, from NCBI). Blastn hits with p < 10^-5^ were considered positive and incorporated to the set of ancestral CNEs (aCNEs).

Sequence conservation data for each human aCNE was extracted from the 46-way-placental PhyloP and PhastCons conservation tracks from UCSC table browser ([62], http://genome.ucsc.edu/cgi-bin/hgTables?command=start). PhyloP score provides a measure of the conservation or divergence of a particular alignment position (negative values indicate purifying selection, whereas high positive scores suggest accelerated evolution). PhastCons scores indicate the degree of conservation of a nucleotide position in a multi-species alignment (from fully conserved (=1) to not conserved (=0)). For both PhyloP and PhastCons we calculated the average score for each CNE and performed ANOVA tests between CNEs conserved and lost in zebrafish. In addition, we compared the proportion of highly conserved or diverged positions within each of the two groups using Chi-squared tests.

### Identification of enhancers target genes and comparison of gene expression patterns

To associate the tested CNEs to their respective target genes, we first searched for genomic regulatory blocks (GRB) based on conserved synteny across vertebrate species (Additional file 9 A). Some of these GRBs were previously defined [63] and for the others we have used Synorth software ([64]; http://synorth.genereg.net/). Then, one or few target genes from these GRBs containing the respective CNE were selected based on consistency of CNE and gene expression patterns in mice [26,65-68] (Additional file 9B and C, E and F). For these cases, we searched for available *in situ* hybridization (ISH) and/or RT-PCR gene expression data (EMAGE [66]; Eurexpress [67]; Genepaint [68]; GXD [65]). Once we have confidently established the association between the CNE and the endogenous gene in mammals, we investigated the gene expression in the orthologous gene of zebrafish in available ISH database [69] (Additional file 9D and G; Additional file 10). For some genes with no available expression data (*gsx2* (ENSDARG00000043322) and *znf423* (ENSDARG00000059707)), we performed whole mount ISH in zebrafish embryos (Additional file 11). In the case of CNEs that have diverged beyond recognition/lost in zebrafish and for which there are two paralogs in this species, we discarded the element if the candidate genes had discrepant expression. For the set of confident gene-CNE associations in both species, we then annotated the presence or absence of gene expression in the homologous mouse and zebrafish anatomical domains as per CNEs. This way we were able to build a 4-way comparison for 26 CNEs with presence/absence of signal in transgenic mice and zebrafish and the endogenous mouse and zebrafish target genes (Additional file 3).

In addition, we found that, for four of the studied human CNEs, the target genes have no corresponding ortholog in zebrafish (Hs426-Pou3f4, Hs240 and Hs752-TLE1 and Hs312-TLE4). Consistently, none of these four CNEs could be identified in the genome of zebrafish or of any other available teleost, despite their presence in shark and tetrapod genomes. However, the associated genes could be identified in the other four teleost genomes, suggesting that these are zebrafish-specific gene losses. The most plausible evolutionary scenario, then, is that first the CNEs were lost in the lineage of teleost fish and then the gene orthologs were lost in zebrafish. Interestingly, when these regulatory modules are tested for enhancer activity in zebrafish they are still able to reproduce part of the mice transgenic expression patterns (Additional file 12).

### Zebrafish whole-mount *in situ* hybridization

Antisense RNA probes were prepared from cDNAs using digoxigenin (Roche) as label. cDNAs were amplified by RT-PCR from total RNA extracted from different developmental stages of zebrafish embryos, treated with DNAse, and processed as described [20,70]. Coding regions of genes were amplified using the following primers:

gsx2FW:5^′^- ACTGGGCACTGCGCAAGTTCTT-3^′^

gsx2RV: 5^′^-CTTTGCCTTCTTTCTTGTGCTTAACGC - 3^′^

znf423FW: 5^′^-TGTGTGATTACTGTGAGGAAACATTC -3^′^

znf423RV: 5^′^-GCTGTCACCAGTTACCCTATGG-3^′^

The amplification products were subcloned in pGEMT-Easy (Promega) and sequenced. Zebrafish specimens were prepared, hybridized and stained as described [71].

## Competing interests

The authors declare that they have no competing interests.

## Authors' contributions

MI, JLGS and JB conceived and designed the experiments. AAC, MI and JB performed the experiments. AAC, MI, HBF and JB analysed the data. AV and LP contributed reagents and materials. HBF, AV and LP revised the paper. MI, JLGS and JB wrote the paper. All authors read and approved the final manuscript.

## Supplementary Material

Additional file 1 Comparison of expression patterns in mice and zebrafish driven by the tested CNEs. Click here for file

Additional file 2 Comparison of expression patterns in mice and zebrafish driven by ancestral CNEs. Click here for file

Additional file 3 4 way comparison of expression patterns driven by CNEs and corresponding target genes in zebrafish and mice. Click here for file

Additional file 4 Graph representing the number of times expression is detected per anatomical domain, in mouse (orange) or zebrafish (blue) enhancer activity assays. Click here for file

Additional file 5 **Comparison of transient and stable transgenesis in zebrafish for the enhancer activity assay of the Hs200 CNE.** A) Expression driven by Hs200 in 24hpf transient transgenic embryos is mostly detected in the forebrain. B) A stable transgenic line for the Hs200 CNE show strong expression in the forebrain but also a weaker reproducible expression in the midbrain and hindbrain in 24hpf embryos. Click here for file

Additional file 6 Comparison of expression patterns in mice and zebrafish driven by mammal sequences reported in other datasets. Click here for file

Additional file 7 Summary of expression patterns driven by CNEs in zebrafish and mice enhancer assays. Click here for file

Additional file 8 **GFP expression in a representative stable transgenic line for each CNE.** Images of GFP expression from a representative line for each CNE in 48hpf stable transgenic embryos. Click here for file

Additional file 9 **Synteny, enhancer activity in mice and target gene expression in mice and zebrafish for the Hs215 and Hs335 CNEs.** A) Relative position of Hs215 and Hs335 CNEs and their respective target genes isl1 and ntm. Hs335 is not detected by alignment in the zebrafish genome. B) Expression driven by the Hs215 enhancer in the eye, spinal cord, dorsal root ganglia and cranial nerve is shared by its target gene, islt1, in mice (C; inset is part of another section from the same embryo sowing expression in the eye) and zebrafish (D). E) Expression driven by the Hs335 enhancer in the spinal cord and limbs is shared with its corresponding target gene ntm (F; inset is part of another section from the same embryo sowing expression in the limb) but it does not coincide with the ntm ortholog in zebrafish (G). Click here for file

Additional file 10 Genomic location of tested CNEs and respective target genes. Click here for file

Additional file 11 **In situ hybridization performed in 24hpf zebrafish embryos for gsx2 and znf423 genes.** A) gsx2 expression is detected in hindbrain and forebrain at 24hpf and 48hpf (B). C) At 22hpf znf423 gene is expressed in the forebrain, hindbrain, eye and spinal cord. D) At 48hpf znf423 gene is detected in the forebrain, midbrain, hindbrain and eye. Click here for file

Additional file 12 **Enhancer activity of CNEs absent from the lineage of teleost fishes in mice and in zebrafish.** A and B) Expression of Hs240 is shared by zebrafish and mice in the forebrain, being singularly expressed in the zebrafish hindbrain. C and D) The Hs426 enhancer shows similar expression in mice and zebrafish (otic vesicle, forebrain and hindbrain). E and F) A species specific expression of the Hs312 enhancer is observed in the hindbrain and midbrain of mice (E) being shared by zebrafish (F) in the spinalcord, limbs and forebrain. G and H) The expression of the H752 enhancer is shared by mice and zebrafish in muscle being mice specific for the dorsal root ganglia, trigeminal ganglion and spinal cord. Click here for file
