# Supplementary figures and images for: Differences in enhancer activity in mouse and zebrafish reporter assays are often associated with changes in gene expression
Source: BMC Genomics. 2012 Dec 19;13:713. doi: 10.1186/1471-2164-13-713 (PMC3541358; doi:10.1186/1471-2164-13-713)

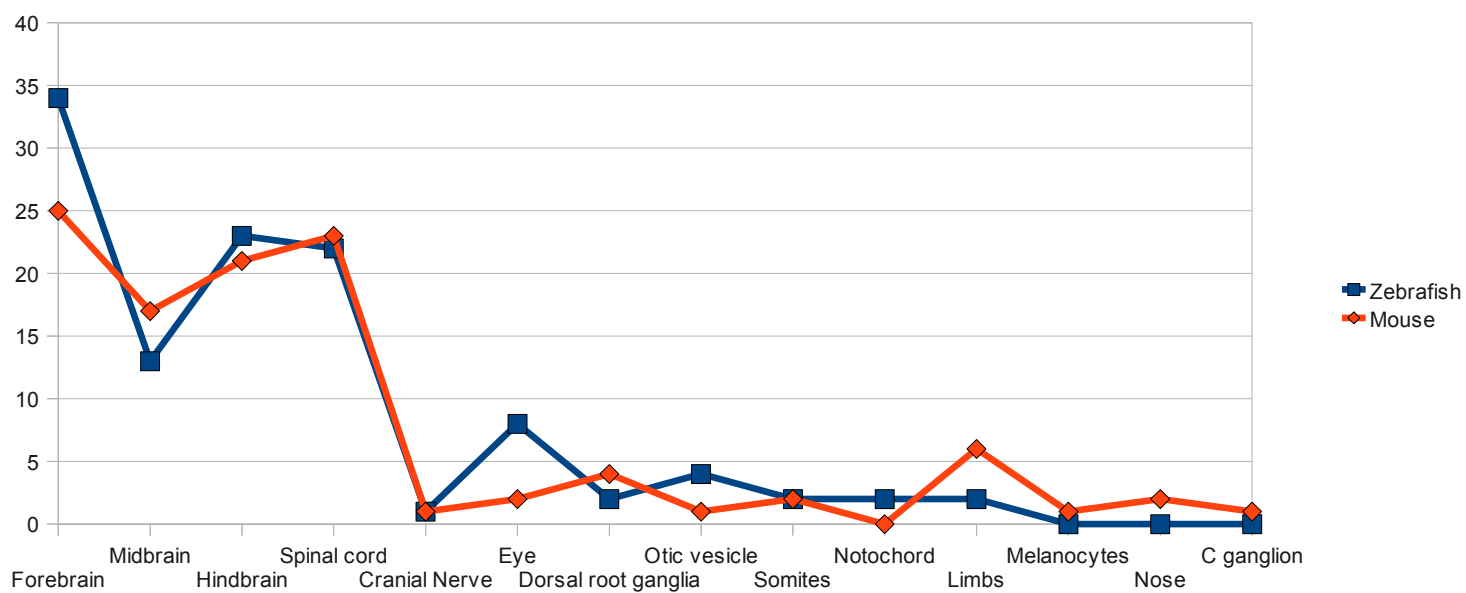

Supplement: Additional file 4 — Graph representing the number of times expression is detected per anatomical domain, in mouse (orange) or zebrafish (blue) enhancer activity assays. [file 1471-2164-13-713-S4.pdf]

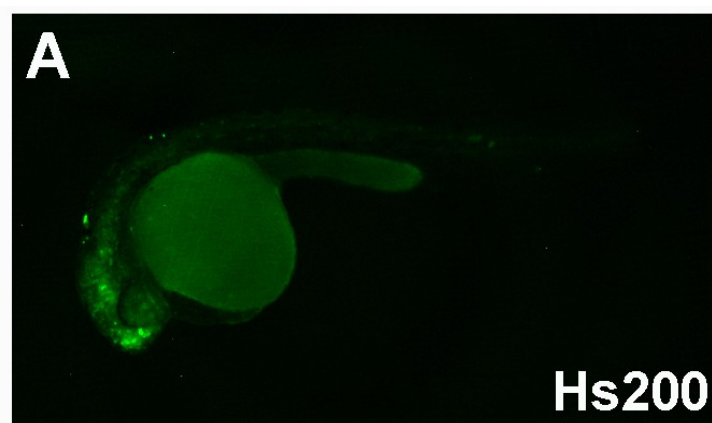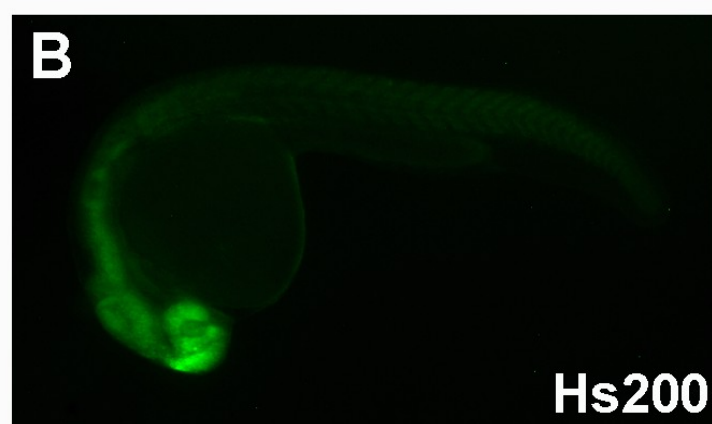

Supplement: Additional file 5 — Comparison of transient and stable transgenesis in zebrafish for the enhancer activity assay of the Hs200 CNE. A) Expression driven by Hs200 in 24hpf transient transgenic embryos is mostly detected in the forebrain. B) A stable transgenic line for the Hs200 CNE show strong expression in the forebrain but also a weaker reproducible expression in the midbrain and hindbrain in 24hpf embryos. [file 1471-2164-13-713-S5.pdf]

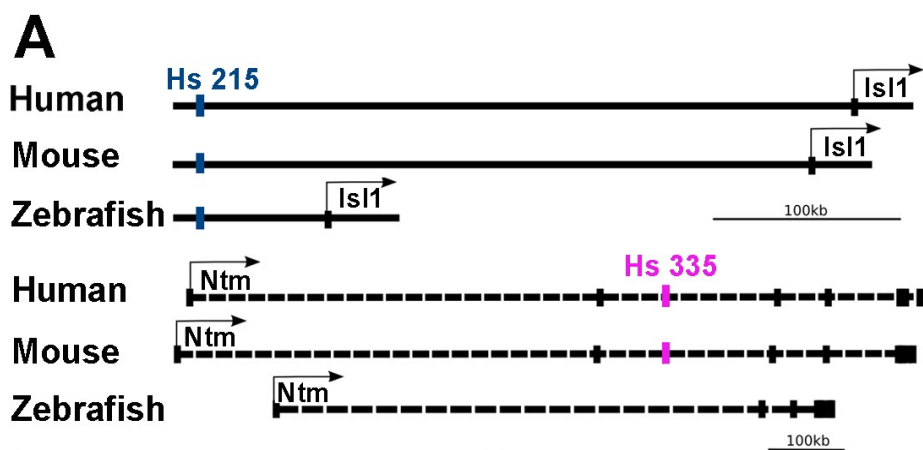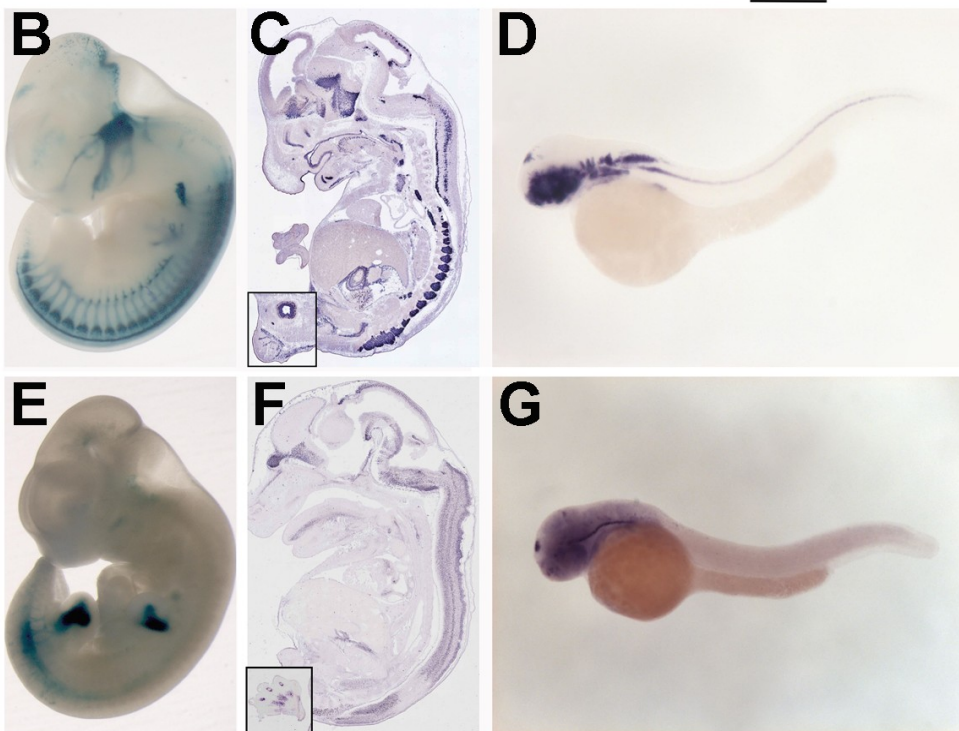

Supplement: Additional file 9 — Synteny, enhancer activity in mice and target gene expression in mice and zebrafish for the Hs215 and Hs335 CNEs. A) Relative position of Hs215 and Hs335 CNEs and their respective target genes isl1 and ntm. Hs335 is not detected by alignment in the zebrafish genome. B) Expression driven by the Hs215 enhancer in the eye, spinal cord, dorsal root ganglia and cranial nerve is shared by its target gene, islt1, in mice (C; inset is part of another section from the same embryo sowing expression in the eye) and zebrafish (D). E) Expression driven by the Hs335 enhancer in the spinal cord and limbs is shared with its corresponding target gene ntm (F; inset is part of another section from the same embryo sowing expression in the limb) but it does not coincide with the ntm ortholog in zebrafish (G). [file 1471-2164-13-713-S9.pdf]

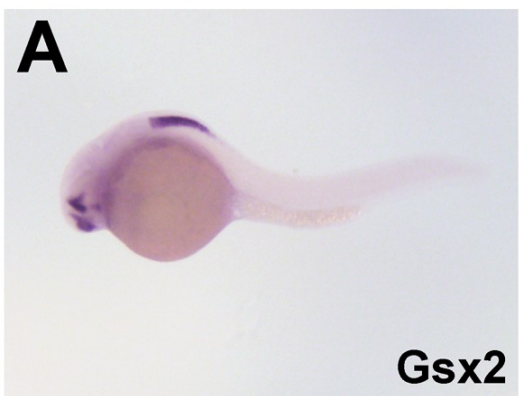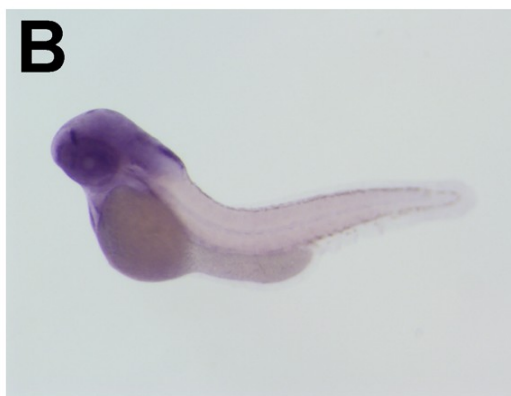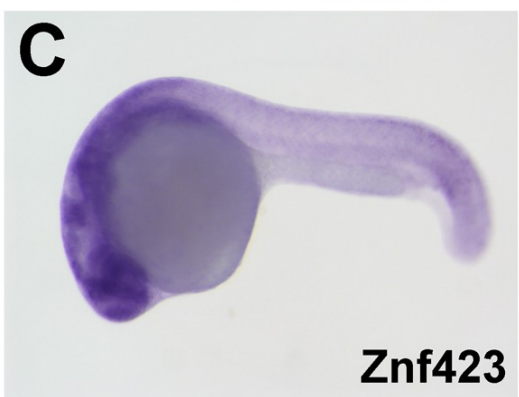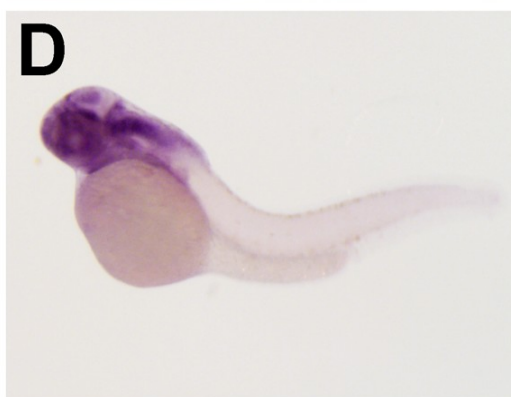

Supplement: Additional file 11 — In situ hybridization performed in 24hpf zebrafish embryos for gsx2 and znf423 genes. A) gsx2 expression is detected in hindbrain and forebrain at 24hpf and 48hpf (B). C) At 22hpf znf423 gene is expressed in the forebrain, hindbrain, eye and spinal cord. D) At 48hpf znf423 gene is detected in the forebrain, midbrain, hindbrain and eye. [file 1471-2164-13-713-S11.pdf]

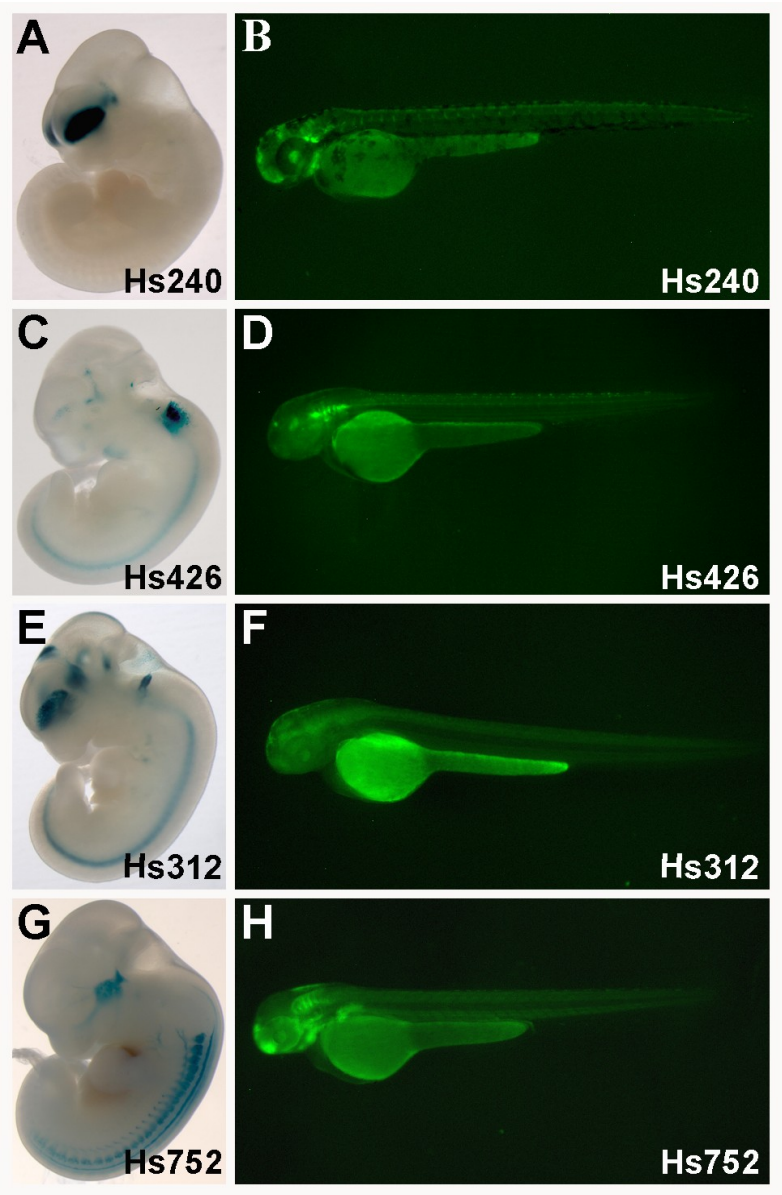

Supplement: Additional file 12 — Enhancer activity of CNEs absent from the lineage of teleost fishes in mice and in zebrafish. A and B) Expression of Hs240 is shared by zebrafish and mice in the forebrain, being singularly expressed in the zebrafish hindbrain. C and D) The Hs426 enhancer shows similar expression in mice and zebrafish (otic vesicle, forebrain and hindbrain). E and F) A species specific expression of the Hs312 enhancer is observed in the hindbrain and midbrain of mice (E) being shared by zebrafish (F) in the spinalcord, limbs and forebrain. G and H) The expression of the H752 enhancer is shared by mice and zebrafish in muscle being mice specific for the dorsal root ganglia, trigeminal ganglion and spinal cord. [file 1471-2164-13-713-S12.pdf]
