# Supplementary material for: Differences in enhancer activity in mouse and zebrafish reporter assays are often associated with changes in gene expression
Source: BMC Genomics. 2012 Dec 19;13:713. doi: 10.1186/1471-2164-13-713 (PMC3541358; doi:10.1186/1471-2164-13-713)
Supplement: Additional file 8 — GFP expression in a representative stable transgenic line for each CNE. Images of GFP expression from a representative line for each CNE in 48hpf stable transgenic embryos. [file 1471-2164-13-713-S8.pdf]

CNEs	Zebrafish 48hpf
Hs 123	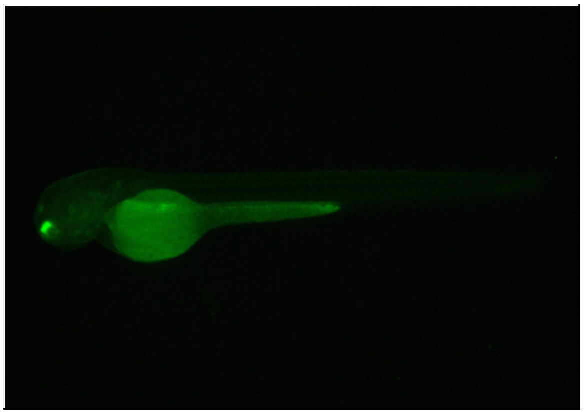
Hs 124	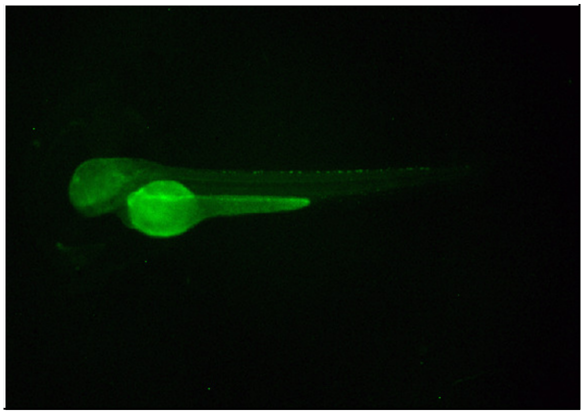
Hs 194	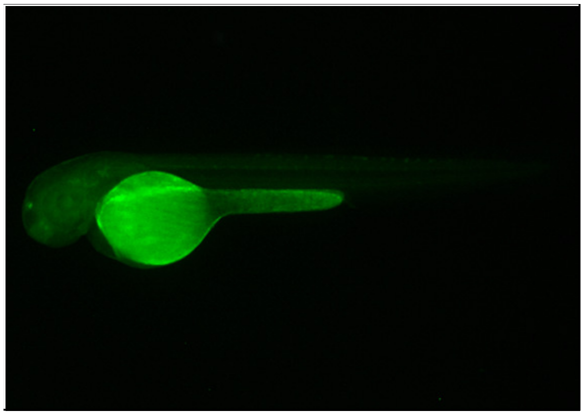

**Hs 200**	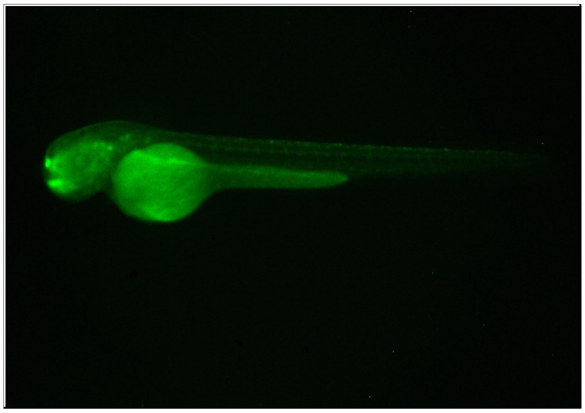
**Hs 215**	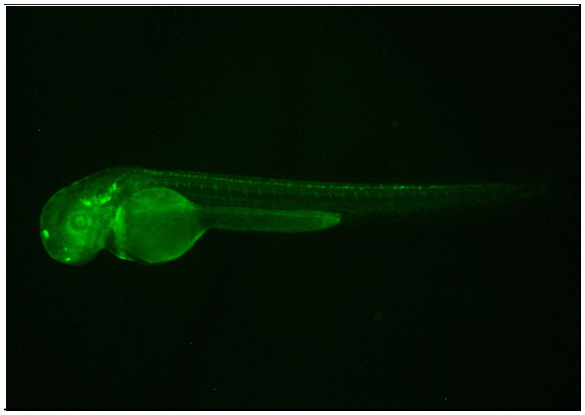
**Hs 240**	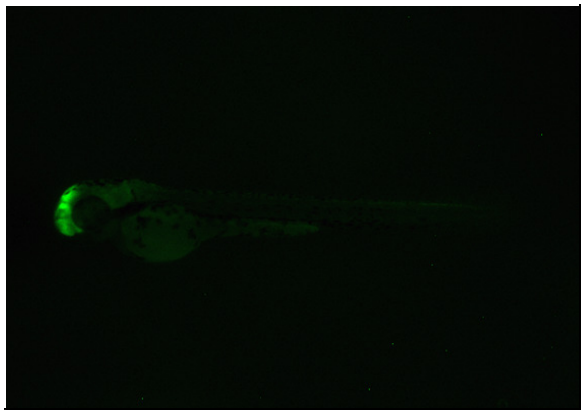
**Hs246**	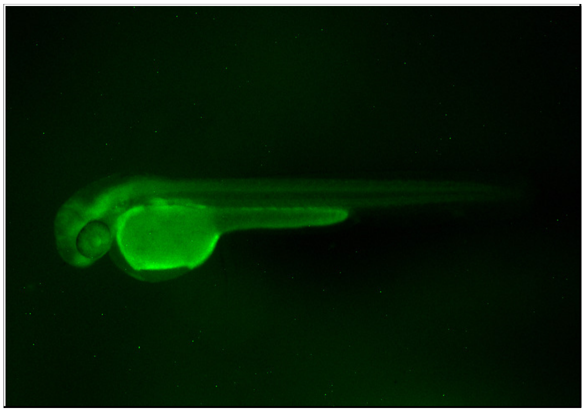

**Hs 259**	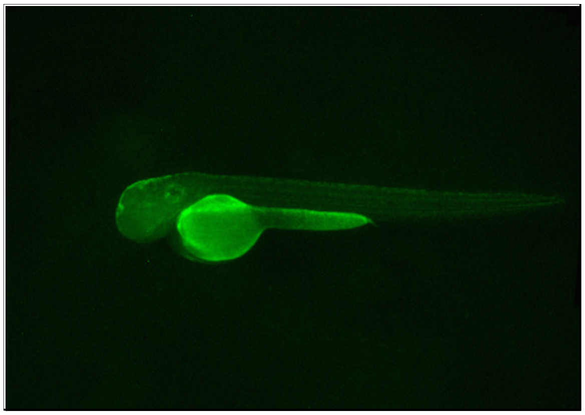
**Hs 260**	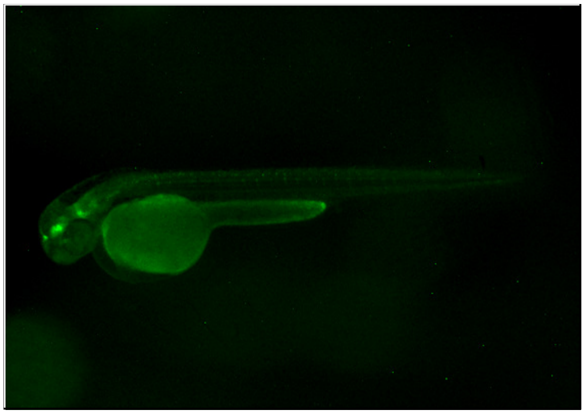
**Hs 266**	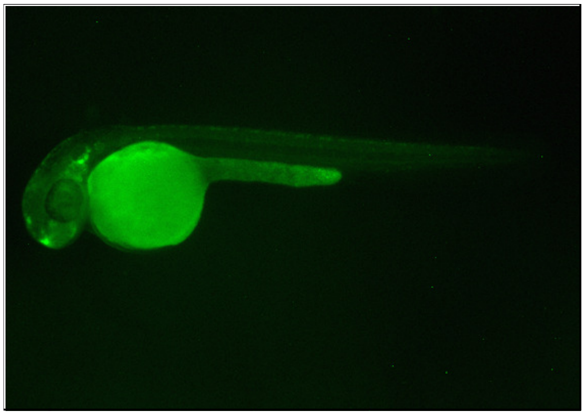
**Hs 267**	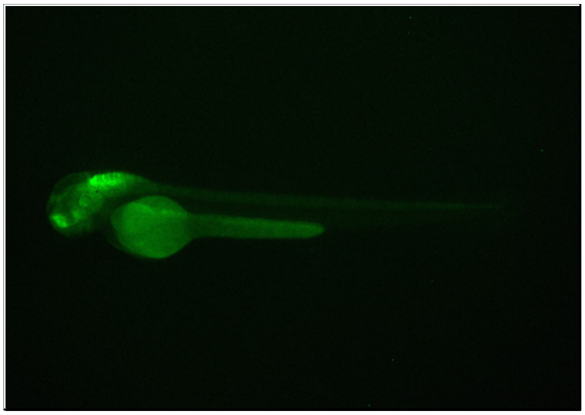

**Hs 271**	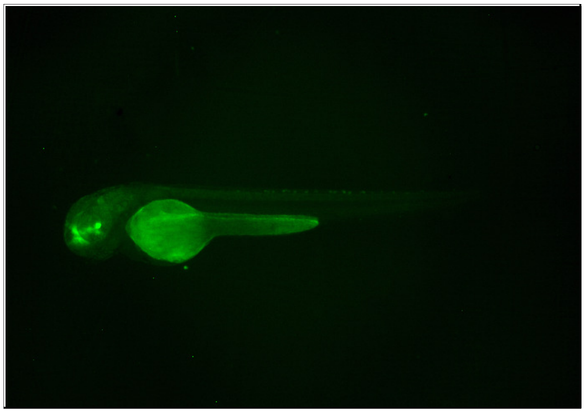
**Hs 278**	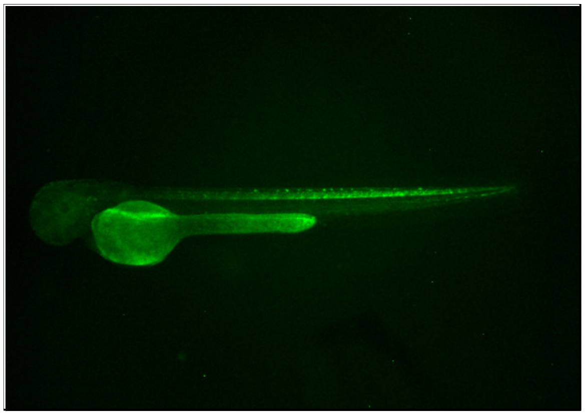
**Hs 284**	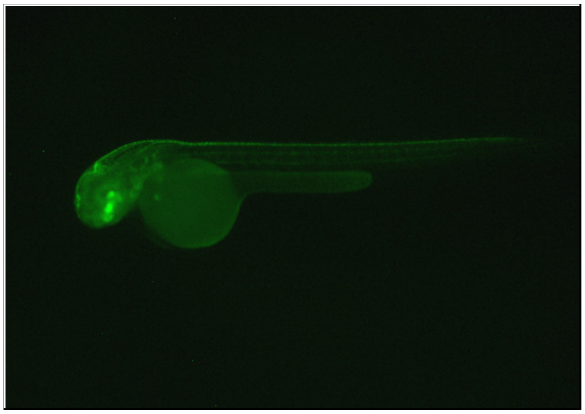
**Hs 312**	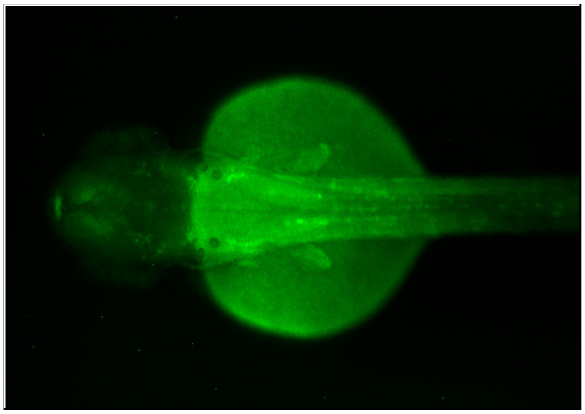

**Hs 327**	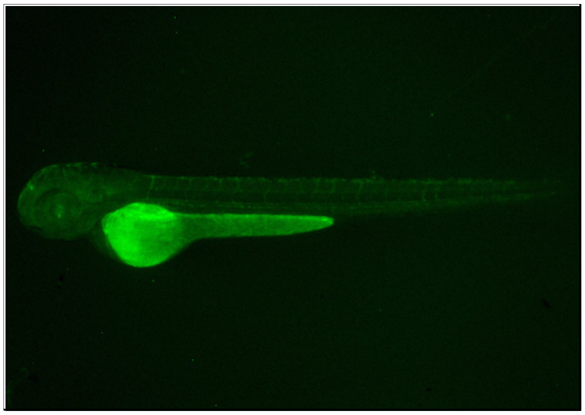
**Hs 335**	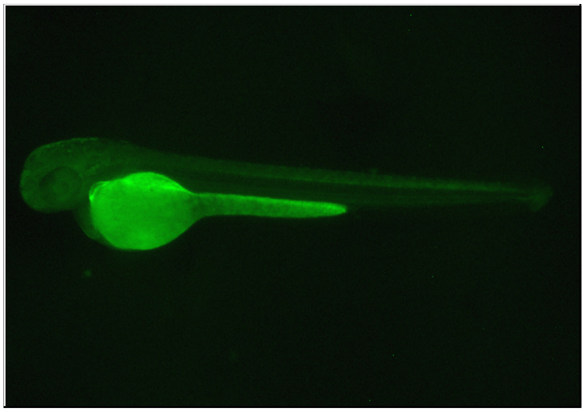
**Hs 382**	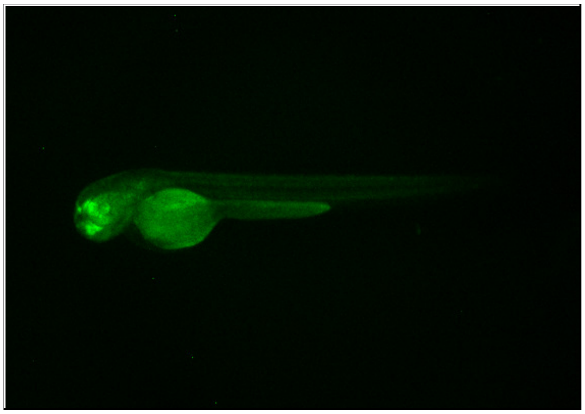
**Hs 411**	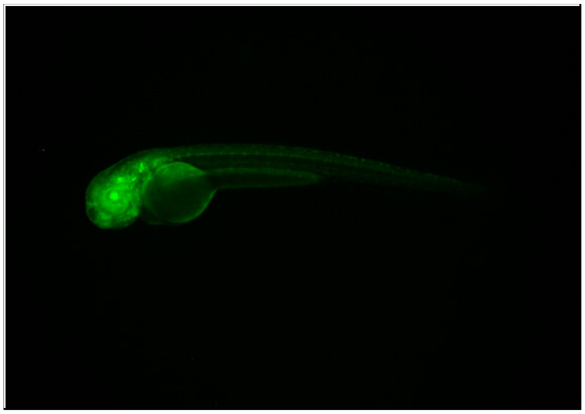

**Hs 422**	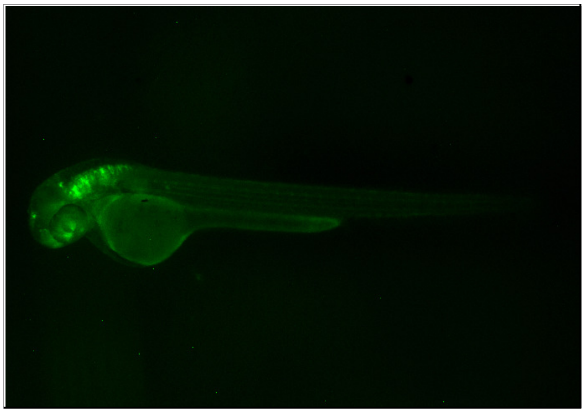
**Hs426**	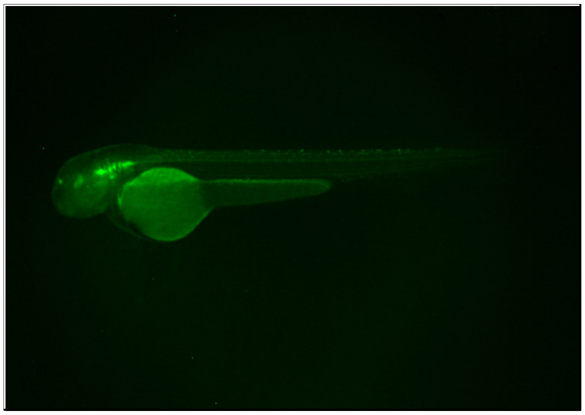
**Hs 428**	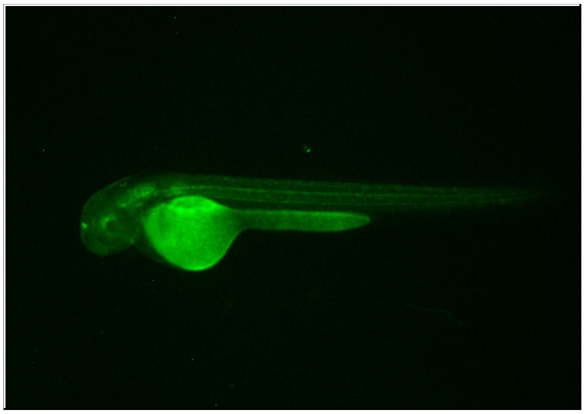
**Hs 567**	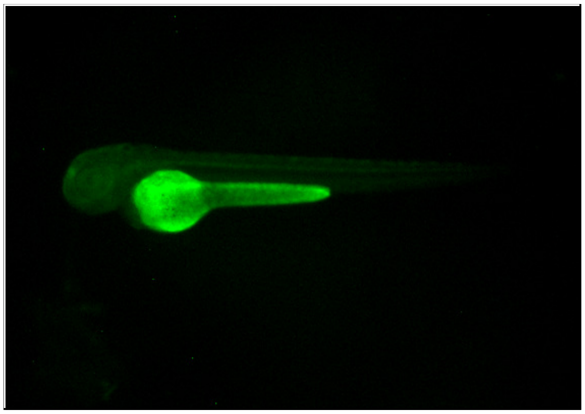

**Hs 595**	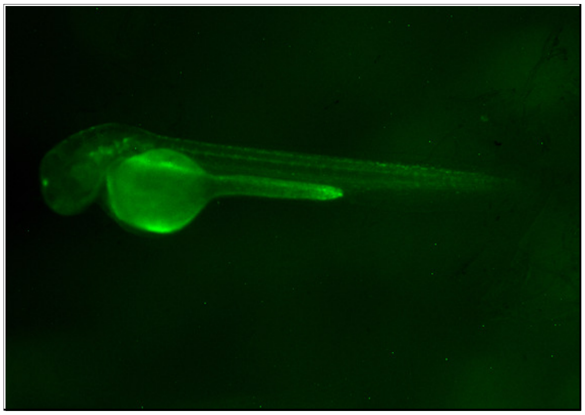
**Hs 608**	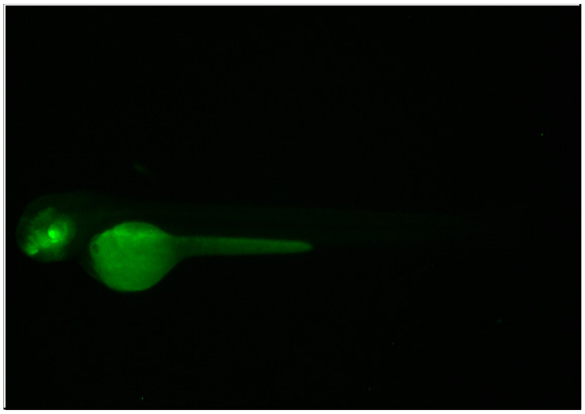
**Hs 611**	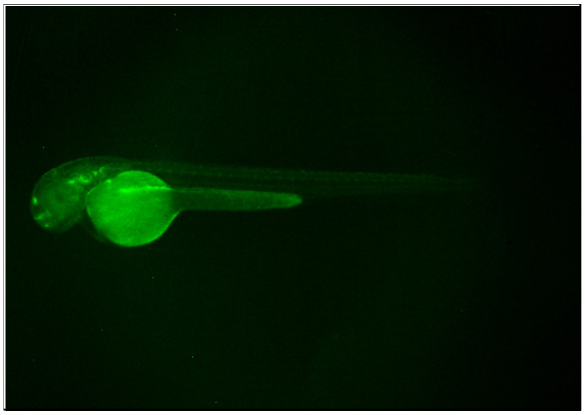
**Hs 669**	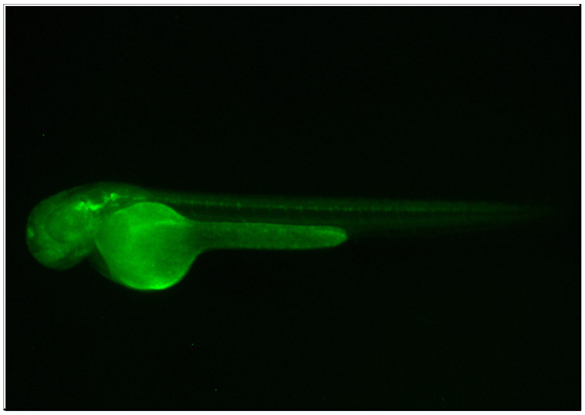

**Hs 671**	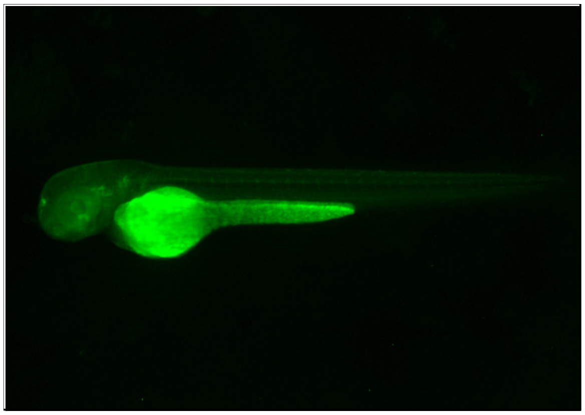
**Hs 672**	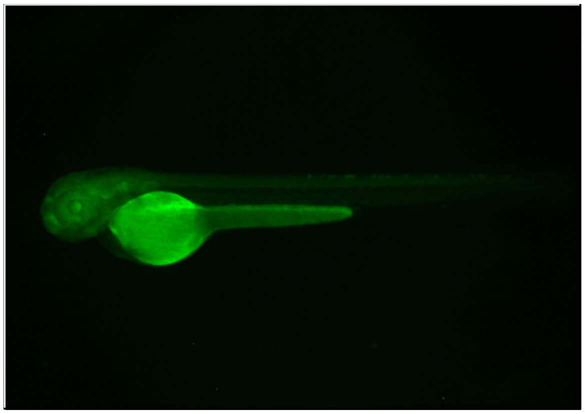
**Hs 687**	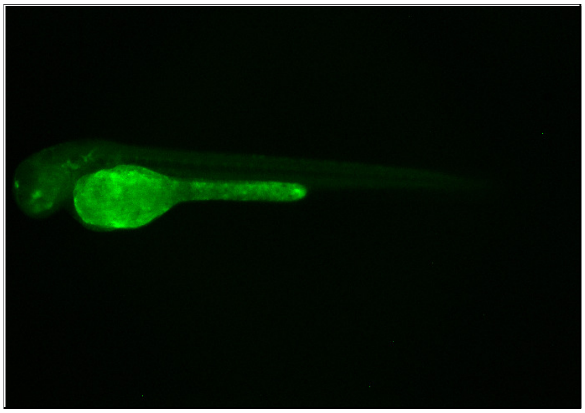
**Hs 688**	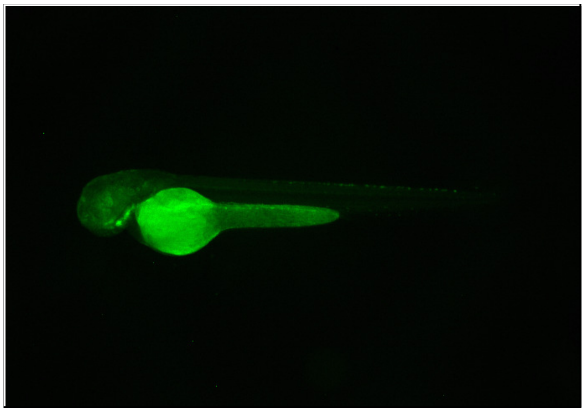

**Hs 699**	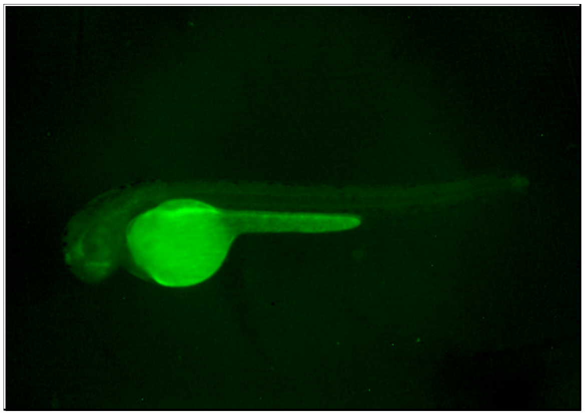
**Hs 752**	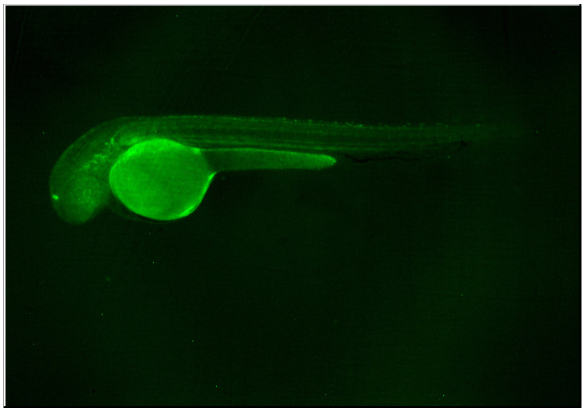
**Hs 755**	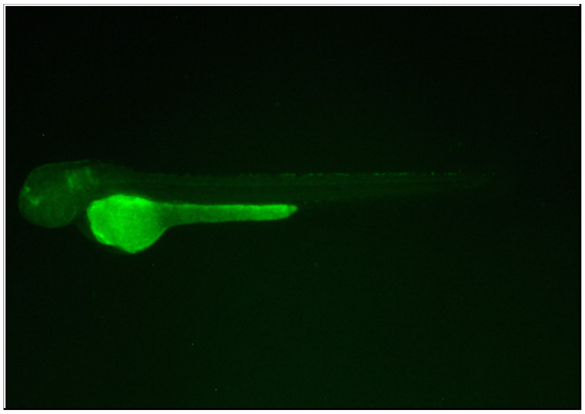
**Hs 774**	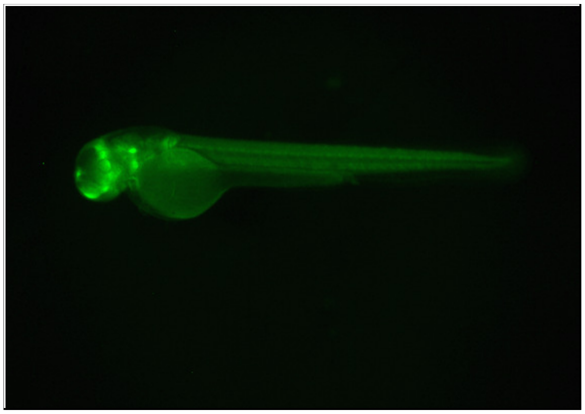

**Hs 793**	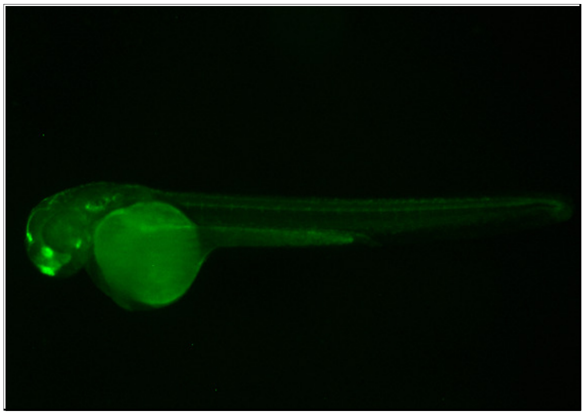
**Hs 799**	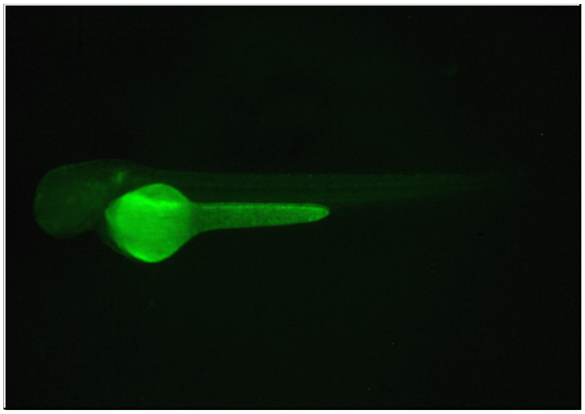
**Hs 807**	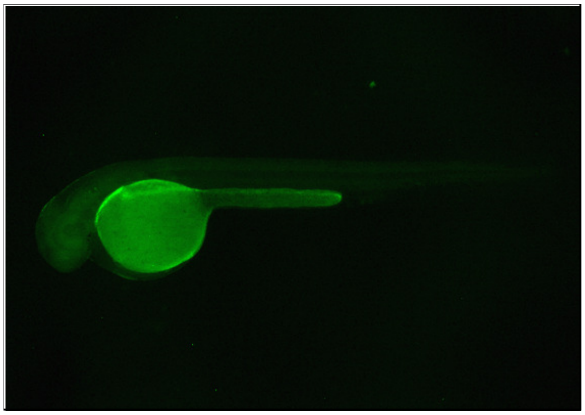
**Hs 816**	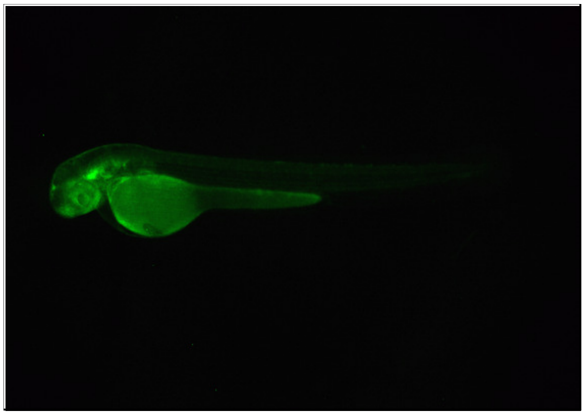

**Hs886**	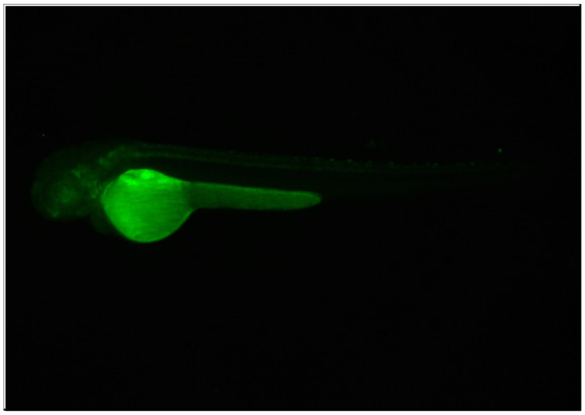
